# Pigmentation and the Diagnosis of Addison's Disease

**Published:** 1908-09-19

**Authors:** 


### Medicine.

### PIGMENTATION AND THE DIAGNOSIS OF ADDISON'S DISEASE.

Abnormal pigmentation of the skin and mucous membranes is a prominent feature of many cases of Addison's disease; and if the pigmentation is known to have developed more or less recently in a person who has other symptoms compatible with the disease, it very often constitutes the clinching point in the diagnosis. More stress is usually laid upon the presence of pigmented spots and areas on the mucuous surfaces of the lips and cheeks than upon similar spots in the external skin; but there are cases of Addison's disease in which no abnormal pigmentation of mucous membranes is to be detected, and there are also certain other conditions in which buccal pigmentation may be present.

Microscopically the pigment in the skin is found in the cells of the stratum Malpighii, the more superficial layers of the epidermis being almost or quite free from it. The dermis shows a few "carrier" pigmented cells which are regarded as conveying the pigment from the blood vessels of the dermis to the stratum Malpighii. The pigment in question is the same as that which is normally present in the deeper layers of the skin. It does not contain iron, so that if it is indirectly derived from hæmoglobin as some think it has undergone considerable metabolism before or during its transmission by the carrier cells.

In the case of mucous membranes the abnormal pigmentation is never general; it requires local friction, hyperæmia, or irritation for its development. Although there is a stratum Malpighii in the tongue and buccal mucosa, there is little or no pigment in it, and microscopic sections through the pigmented spots in Addison's disease show that the accumulation is in carrier cells in the corium rather than in the stratum mucosum as in the case of the skin. When there is pigment in the stratum mucosum the granules are between the cells, not within them.

The abnormal pigmentation of both the skin and the mucous membranes is very variable in extent and degree. In most cases it is one of the later symptoms of the disease, occurring only some time after the extreme asthenia, the attacks of vomiting, and possibly of syncope, have been complained of. It may take the form of a general browning of the skin, with no particular spots or speckles. More often there are special deposits of pigment in the parts which normally contain most pigment—the fingers and toes, the face, the areolæ of the nipples, the axillæ, the linea alba, the genital organs, and the groins. In addition to this one expects to find small spots and speckles that are almost black scattered about over the limbs or trunk over and above the general bronzing. In some cases these individual pigmented spots are a marked feature without any general bronzing, in which case they are usually to be found upon the extensor surfaces of the arms and forearms, on the backs of the hands, and above and below the knees.

Irritation is an important factor in determining the site of pigmentary deposits. Parts of the body which are exposed to friction or pressure show increased pigmentation in an especial degree. Thus the waist where it is compressed by corset or belt, the knee where garters are tied, the shoulders under the braces, the foot or ankle where boots have pressed, and the prominent vertebral spines present a darker hue. Nevertheless, the palms of the hands and the soles of the feet are very rarely involved. The tissue of scars remains unaffected, but the surrounding skin may show an exaggeration of pigmentation. Places that have been blistered recently or irritated by some application such as a turpentine liniment or a mustard plaster, become very brown, even more so than they do in other persons. The regions of the hairy scalp, the beard, and the eyebrows escape.

Irritation is also a very important factor in determining the degree and the site of pigmentation in the mouth. In those who have perfect teeth and who do not smoke pigment may not appear here at all. In those who smoke, on the other hand, or in those who have any lesion of the teeth, there are nearly always dark pigmentary patches on the inner surfaces of the lips and cheeks and on the tongue. On the lips the line where they come in contact is the commonest site; on the cheeks the pigment is seen along the lines corresponding to the two lines of teeth. The tongue may show purplish stains like ink-marks along its free border, usually so distributed as to suggest irritation by the teeth as their exciting cause.

The buccal mucosa is that which is most easily examined; but in many cases there is similar pigmentation near the anal orifice, or upon the glans penis and within the external urinary meatus. The nasal mucosa and the conjunctivæ have also been noted as presenting spots of pigment in a few instances. Generally speaking pigmentation, though suggesting Addison's disease, is not of itself, apart from constitutional symptoms, sufficient to warrant a positive diagnosis. In the absence of pigmentation it is very difficult to be sure of Addison's disease. In its presence, however, asthenia, vomiting and syncopal attacks—particularly grave asthenia—are strongly suggestive of Addison's disease.

In regard to the existence of buccal pigmentation in other affections than Addison's disease, it will almost suffice to enumerate the conditions under which it has been observed. Lascars and negroes have an abundance of buccal pigmentation, and when there is any strain of this blood in the patient it may show itself within the mouth when the general condition of the body may not appear other than that of a pure-blooded white man. The difficulty in diagnosis will be increased if the facts of the case are withheld by the patient, as may very likely happen. There is little difficulty in distinguishing between a person of decidedly coloured extraction and a patient who is deeply pigmented in Addison's disease, for in the latter the pigment on the fingers throws the unpigmented nails into very marked relief, making them appear almost unnaturally white.

There are a few normal persons who present pigment patches within the cheeks and on the mucous surface of the lips, particularly when these parts have been long subjected to irritation by bad teeth or by pipe stems and tobacco smoke. The tendencies to this pigmentation is apt to increase during long illness, and one sees cases of phthisis now and then in which, if the buccal mucosa alone were relied on, tuberculosis of the suprarenal glands as well as of the lungs would be diagnosed, and yet at autopsy the suprarenals turn out to be perfectly healthy.

Finally there are occasional cases in which arsenic has been given over long periods, and in which the pigmentation affects not only the skin but also the buccal mucosa. This might happen in pernicious anæmia, for example, or Hodgkin's disease, chorea, psoriasis, lichen planus, and other diseases that are treated by arsenic.

